# LIN28B promotes differentiation of fully transformed AML cells but is dispensable for fetal leukemia suppression

**DOI:** 10.1038/s41375-024-02167-0

**Published:** 2024-02-06

**Authors:** Yanan Li, Jonny Mendoza-Castrejon, Riddhi M. Patel, Emily B. Casey, Elisabeth Denby, David Bryder, Jeffrey A. Magee

**Affiliations:** 1 Division of Hematology and Oncology, Department of Pediatrics, Washington University School of Medicine, 660 S. Euclid Ave, St. Louis, MO 63110 USA; 2 Division of Molecular Hematology, Department of Laboratory Medicine, Lund University, 221 84 Lund, Sweden

**Keywords:** Acute myeloid leukaemia, Developmental biology

## To the Editor:

Infant leukemias present within the first year of life as either acute lymphoblastic leukemia (ALL) or acute myeloid leukemia (AML). A majority are driven by *KMT2A/MLL* rearrangements (henceforth MLLr) that arise before birth [1, 2]. Aside from MLLr, infant leukemias harbor few additional cooperating mutations [3, 4]. Thus, the threshold of genetic change required for infant leukemia initiation, and the time required to cross the threshold, are both low when compared to MLLr adult leukemias. These observations suggest that MLLr transform fetal hematopoietic progenitors more efficiently than adult progenitors.

Prior studies in both mouse and human systems have supported the idea that MLLr transform fetal/neonatal progenitors more efficiently than adult progenitors [5–7], but they have also raised interesting caveats that portend therapeutic opportunities. For example, we recently used an inducible transgenic mouse model of MLL::ENL-driven AML to show that the MLL::ENL initiates leukemogenesis most efficiently when it is induced immediately after birth rather than before [5]. Fetal MLL::ENL induction did cause AML, but with longer latency and lower penetrance than postnatal induction. This result suggests that one or more fetal-specific gene products might impede transformation. Indeed, co-expression of a fetal master regulator, *Lin28b*, suppressed rather than accelerated transformation [5].

Several lines of evidence support the notion that *Lin28b* suppresses leukemic transformation before birth, but not afterwards. *Lin28b* is highly expressed prior to birth, and expression declines postnatally concordant with a transition from fetal to adult transcriptional states [8, 9]. While *Lin28b* encodes an RNA binding protein best known for its ability bind and inhibit *let-7* microRNA (miR) precursors [10], Eldeeb et al. recently showed that LIN28B suppresses AML initiation independently of its *let-7* binding activity [11]. Instead, they found that LIN28B binds the *MYBBP1A* transcript to stabilize translation. MYBBP1A then binds the MYB transcription factor to prevent target gene expression and leukemogenesis [11]. These observations implicated *Lin28b* as a fetal developmental regulator that can impede leukemogenesis. However, they did not test whether conditional deletion of *Lin28b* in fetal progenitors potentiates AML initiation, as one would expect if it were the sole effector of fetal protection, nor did they test whether LIN28B could impede the growth of fully transformed AML cells. The latter point is important because it raises the possibility of reprogramming developmental states, by reinstating all or part of a fetal transcriptional program, to eradicate AML.

**Fig. 1:**
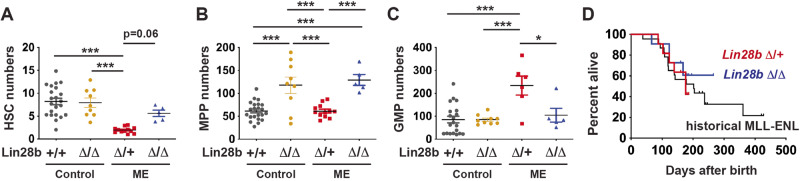
*Lin28b* deletion rescues HSC depletion and GMP expansion in Tet-OFF MLL::ENL mice, but it does not accelerate leukemogenesis. **A**–**C** HSC, MPP and GMP total cell numbers per newborn (P0) liver of control (Cre−), *Lin28b*^*f/f*^*; Vav1-cre* (*Lin28b*^*Δ/Δ*^), Tet-OFF MLL::ENL (ME) and *Lin28b*^*Δ/Δ*^*; ME* mice. For all panels, error bars indicate standard deviation. *n* = 5–22, *p < 0.05, ***p < 0.001 by one-way ANOVA with Holm-Sidak post hoc test. **D** Survival of Tet-OFF MLL::ENL mice of the indicated *Lin28b* genotypes after fetal MLL::ENL induction. *n* = 11–12. A previously described survival curve for Tet-OFF MLL::ENL mice is included for reference purposes [5].

To test whether *Lin28b* is required to impede AML initiation prior to birth, we crossed a previously described Tet-OFF MLL::ENL model (*Vav1-Cre; Rosa26*^*LoxP-STOP-LoxP-rtTA*^*; Col1a1*^*TetO_MLL::ENL*^) to *Lin28*^*flox*^ mice to generate Tet-OFF MLL::ENL; *Lin28b*^Δ/Δ^ offspring [5, 12, 13]. The mice were maintained off doxycycline (DOX) to permit fetal MLL::ENL induction at ~embryonic day (E)10.5, concordant with *Vav1*-Cre expression. We measured hematopoietic stem cell (HSC; CD150^+^CD48^−^Lineage-Sca1^+^Kit^+^), multipotent progenitor (MPP; CD150^−^CD48^+^Lineage-Sca1^+^Kit^+^) and granulocyte-monocyte progenitor (GMP; CD150^−^CD105^−^CD16/32^+^Lineage-Sca1^−^Kit^+^) numbers at postnatal day (P)0 as previously described [14]. As in our prior study, MLL::ENL expression depleted HSCs and promoted GMP expansion (Fig. 1A, C). *Lin28b* deletion rescued these phenotypes, suggesting that *Lin28b* helps promote MLL::ENL-driven HSC depletion and granulocyte differentiation prior to birth (Fig. 1A, C). Interestingly, *Lin28b* deletion increased MPP numbers, irrespective of MLL::ENL expression (Fig. 1B). Altogether, *Lin28b* appears to rescue changes in fetal/neonatal hematopoiesis that ensue following fetal MLL::ENL induction.

We next tested whether *Lin28b* deletion could accelerate AML initiation and reduce survival following fetal MLL::ENL induction. If *Lin28b* is a critical effector of fetal protection, then AML initiation should be more rapid and highly penetrant in *Lin28b*^Δ/Δ^ mice than in *Lin28b*^+/Δ^ mice. However, when we plotted survival of Tet-OFF MLL::ENL; *Lin28b*^+/Δ^ and Tet-Off MLL::ENL; *Lin28b*^Δ/Δ^ mice, the curves tracked with a previously described survival curve for Tet-OFF MLL::ENL mice following fetal induction, and there was no significant difference between the genotypes (Fig. 1D) [5]. Furthermore, *Lin28b* deficiency did not recapitulate the highly penetrant, rapid rate of AML initiation that occurs following postnatal MLL::ENL induction [5]. Other tumor suppressors might contribute to the reduced rate/penetrance of AML initiation that follows fetal MLL::ENL induction. Of note, the data do not resolve the possibility that *Lin28b* could enhance or impede leukemogenesis in older mice because the experiment was terminated at ~6 months after birth. However, adult progenitors and MLL::ENL-driven AML cells do not express *Lin28b* transcript (Supplementary Fig. 1), so there is little reason to think that loss of *Lin28b* would impact AML initiation at older ages.

**Fig. 2:**
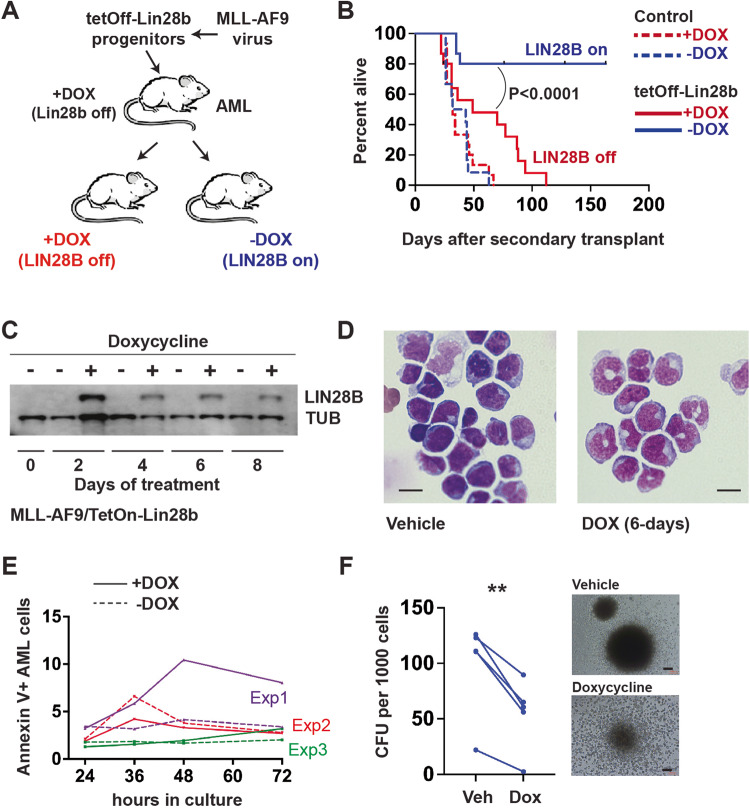
LIN28B suppresses fully transformed AML by promoting differentiation and loss of clonogenicity. **A** Schematic overview of experiment. **B** Survival curves for recipients of 3 independent AML (5 recipients per AML per condition, *n* = 15 total) after LIN28B induction or suppression. Mice transplanted with control AML, which lacked the LIN28B transgene, are indicated with dashed curves. **C** LIN28B and alpha-tubulin (TUB) expression by Western blot in a Tet-ON AML model after the indicated days of DOX treatment. **D** Cytospins showing blast morphology after LIN28B induction. Scale bars indicates 10 μm. **E** Annexin V assays showing percent apoptotic cells (with and without DOX) for 3 independent AML (as indicated by different colors). Paired Student’s *t* tests did not reveal significant differences in apoptosis at any of the time points tested. **F** Colony forming units per 1000 plated AML cells after LIN28B induction. Representative colony morphology is shown to the right. Each paired data point indicates a unique AML. Scale bar indicates 2000 μm. **p < 0.01 by paired Student’s *t* test.

We next tested whether LIN28B can suppress growth of fully transformed AML cells. Most human pediatric AML, including MLLr AML, do not express *LIN28B* or the *let-7* target *HMGA2*, and they express several *let-7* family miR at high levels (Supplementary Fig. 2) [4]. These observations are consistent with the notion that LIN28B antagonizes AML growth, even in leukemias that arise early in life. We transduced *Vav1-Cre; Rosa26*^*LoxP-STOP-LoxP-tTA*^*; Col1a1*^*TetO_Lin28b*^ (Tet-OFF LIN28B) progenitors with MLL::AF9/green fluorescent protein (GFP) retrovirus (Fig. 2A). These progenitors expressed LIN28B in the absence of DOX, and MLL::AF9 was expressed independently of the tet-transactivator. Donor and primary recipient mice were fed DOX chow to suppress LIN28B expression. Primary AML cells were then transplanted into secondary recipients (10 secondary recipients per primary recipient; *n* = 3 independent primary recipients) with half of the recipients maintained on DOX and half deprived of DOX to enable LIN28B expression (Fig. 2A). LIN28B expression significantly extended survival, with 80% of the mice surviving >4 months post-transplant (Fig. 2B). We were unable to detect GFP^+^ AML cells in the bone marrow of most surviving mice (Supplementary Fig. 3). Doxycycline had no effect on survival of a cohort of *Col1a1*^*TetO_Lin28b*^ -negative control mice (Fig. 2B). Altogether, the data show that LIN28B can suppress MLLr AML growth and may even eradicate the disease.

To better understand the fate of LIN28B-expressing AML cells, we switched to a DOX-inducible model—*Vav1-Cre; Rosa26*^*LoxP-STOP-LoxP-rtTA*^*; Col1a1*^*TetO_Lin28b*^ (Tet-ON LIN28B)—to afford more precise temporal control of LIN28B expression. We generated MLL::AF9 AML as before, and we induced LIN28B expression either in vivo by feeding mice DOX, or ex vivo by exposing cultured cells to DOX. LIN28B protein expression was evident after just 2 days of DOX exposure (Fig. 2C), and it caused partial monocytic differentiation by 6 days post-induction (Fig. 2D). In culture, cell numbers remained relatively static across 8 days of assessment, and programmed cell death rates did not increase (Supplementary Fig. 4 and Fig. 2E). However, colony forming potential was severely depleted in 5 biologically independent, LIN28B-expressing AML (Fig. 1F). Colony morphologies showed extensive differentiation, in contrast to the tight round colonies typically observed from MLL::AF9-expressing AML (Fig. 1F) [15]. LIN28B therefore diminishes AML clonogenic potential without inducing apoptosis.

Altogether, our data establish two key points about the role of *Lin28b* in MLLr leukemia suppression. First, they show that *Lin28b* is not the sole effector of fetal leukemia suppression, given that *Lin28b* deletion did not accelerate AML initiation following fetal MLL::ENL induction. Thus, other factors must help account for the slower rate of AML initiation that ensues after fetal MLL::ENL induction as compared to postnatal induction [5]. Additional studies are required to identify these regulators, as they may help explain why fetal leukemias are exceedingly rare despite prenatal mutation acquisition and the relatively quiet genomes of infant leukemias [3, 4]. Second, while LIN28B is not required for fetal AML suppression, the protein is nevertheless sufficient to induce AML differentiation and eradicate the disease. Though this study utilized MLL::AF9- rather MLL::ENL-driven AML as a model for fully transformed AML and LIN28B expression levels were higher in the transgenic models than occurs physiologically (Supplementary Fig. 1), the results are nevertheless consistent with our prior work showing that LIN28B impedes MLL::ENL-driven AML initiation by stabilizing MYBBP1A expression [11]. Future studies should investigate whether leukemia-suppressive effects of LIN28B extend to other drivers of pediatric AML. Furthermore, it will be important to establish whether LIN28B promotes AML differentiation in human patient-derived xenograft models, given potential species differences in LIN28B expression and function. In principle, developmental reprogramming offers a therapeutic strategy that could complement other targeted therapies. Rather than targeting MLL fusion protein complexes directly, therapies that reinstate fetal transcriptional states could mitigate oncogenic programs that drive MLLr leukemias.

### Supplementary information

Supplementary Figures and methods

## Data Availability

The datasets generated during and/or analyzed during the current study are available from the corresponding author on reasonable request. RNA-sequencing data are available in gene expression omnibus (GSE253725).
