# Supplementary material for: LIN28B promotes differentiation of fully transformed AML cells but is dispensable for fetal leukemia suppression
Source: Leukemia. 2024 Feb 6;38(3):648–51. doi: 10.1038/s41375-024-02167-0 (PMC10912017; doi:10.1038/s41375-024-02167-0)
Supplement: Supplementary file 1 — Supplementary Figures and methods [file 41375_2024_2167_MOESM1_ESM.pdf]

**SUPPLEMENTARY INFORMATION**

**Supplementary Figure 1.** ***Lin28b*** **transcript expression in normal hematopoietic progenitors, MLL::ENL-expressing progenitors and AML.**

**Supplementary Figure 2. Expression of LIN28B and let-7 in human pediatric AML.**

**Supplementary Figure 3. Bone marrow evaluation for retention of AML cells after LIN28B induction.**

**Supplementary Figure 4. AML growth kinetics after LIN28B induction.**

**Supplementary methods.**

**Supplemental references.**

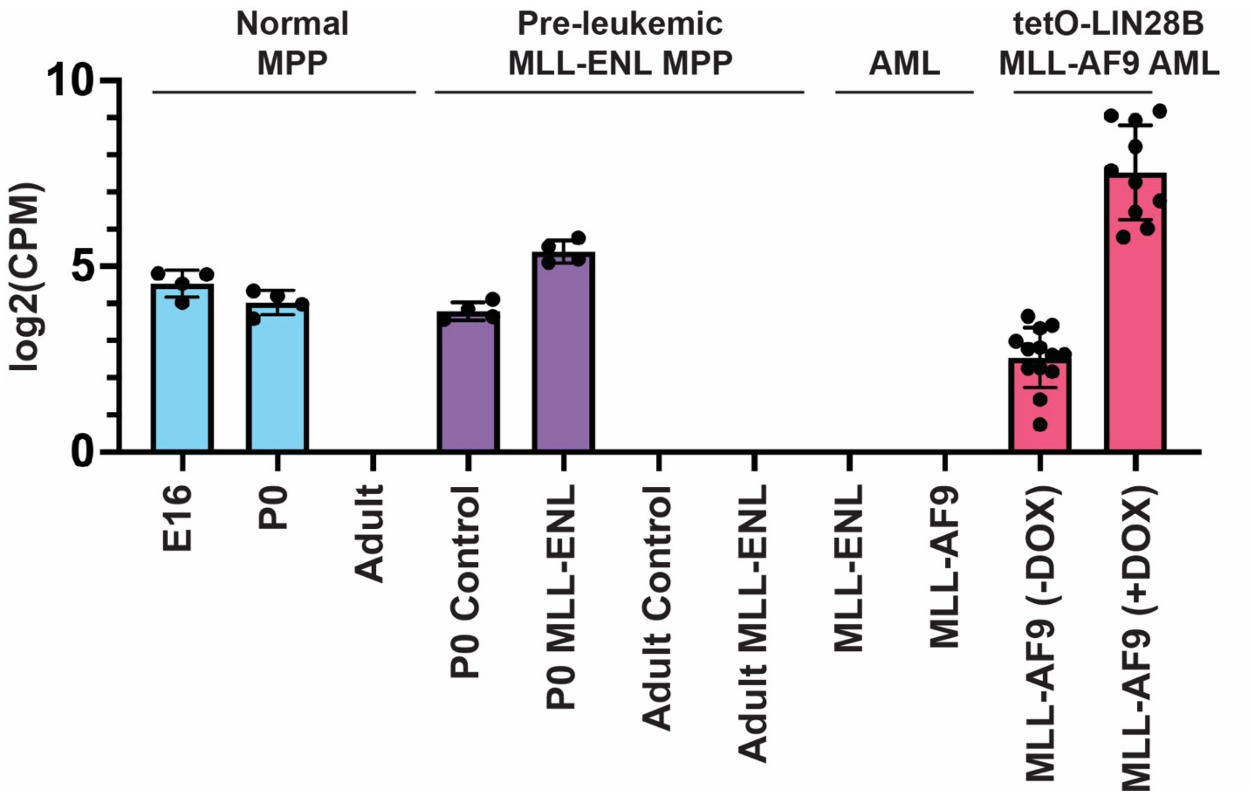


**Supplementary Figure 1. *Lin28b* transcript expression in normal hematopoietic progenitors, MLL::ENL-expressing progenitors and AML.** This plot shows log2(CPM) expression levels for *Lin28b* in a range of contexts. *Lin28b* expression in normal fetal (E16), newborn (P0) and 8-week-old adult MPPs is shown based on data from Ref. 8. *Lin28b* expression in P0 and adult, tet-off MLL::ENL MPPs is shown based on data from Ref. 5. *Lin28b* expression in MLL::ENL and MLL::AF9 AML is shown based on RNA-seq performed in conjunction with this study. Neither MLL::ENL- nor MLL::AF9-driven AML express *Lin28b*. However, when AML is induced on a tet-on LIN28B background, there are detectable levels of expression even in the absence of DOX. Expression increases substantially with DOX treatment.

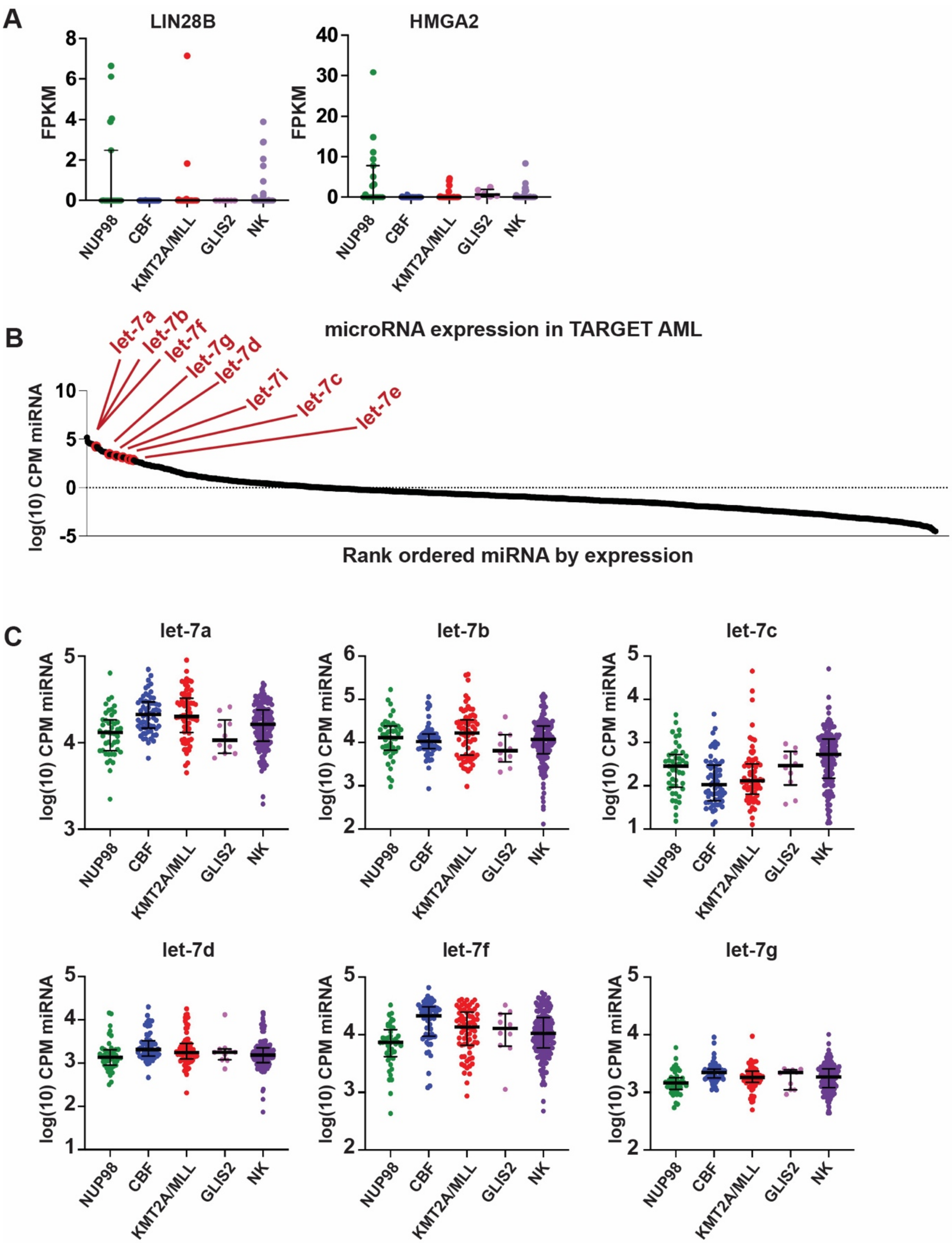
A
LIN28B
FPKM
HMGA2
NUP98
CBF
KMT2A/MLL
GLIS2
NK
B
microRNA expression in TARGET AML
let-7a
let-7b
let-7f
let-7g
let-7d
let-7i
let-7c
let-7e
log(10) CPM miRNA
Rank ordered miRNA by expression
C
let-7a
let-7b
let-7c
let-7d
let-7f
let-7g

**Supplementary Figure 2. Expression of *LIN28B* and *let-7* in human pediatric AML.** (A) *LIN28B* and *HMGA2* expression in pediatric AML based on RNA-seq data collected from the TARGET pediatric AML project (Ref. 4). Expression is indicated for leukemias with *NUP98* rearrangements, core binding factor (CBF) family translocations (*RUNX1::RUNX1T1* or *CBFB::MYH11*), *KMT2A/MLL* rearrangements, *CBFA2T3::GLIS2* rearrangements (GLIS2) or normal karyotype (NK). (B) Expression of *let-7* isoforms in human pediatric AML from the TARGET project. Average expression levels of each miRNA are shown in rank order with the exception of ~10% of the miRNA that showed no expression in any AML. All measured *let-7* isoforms fell within the top 5% of miRNA in terms of expression level. Various *let-7* isoforms are noted in red. (C) Expression of several *let-7* isoforms in pediatric AML with the indicated mutation profiles.

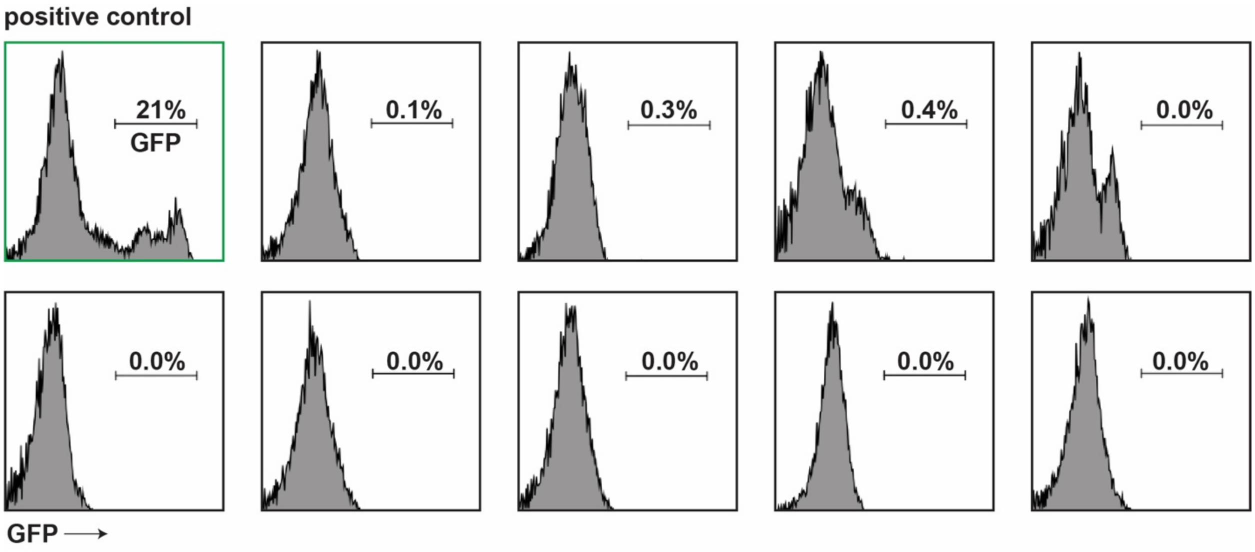


**Supplementary Figure 3. Bone marrow evaluation for retention of AML cells after LIN28B induction.** Representative flow plots are shown for bone marrow harvested from recipient mice that survived after transplantation with MLL::AF9 AML and subsequent LIN28B induction (Figure 2B). AML cells express GFP. A positive control is shown in the top left, and the remaining plots reflect independent recipient mice.

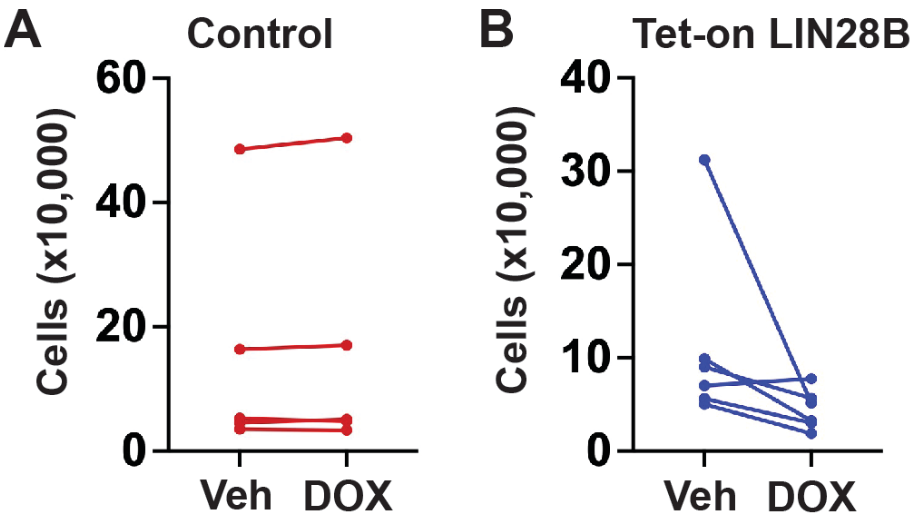


**Supplementary Figure 4. LIN28B does not cause significant depletion of AML cells in short-term culture.** (A, B) Cell counts for 5 control (LIN28B-negative) and 6 Tet-on LIN28B AML cultures derived from MLL::AF9 leukemias. Cultures were initiated with 50,000 GFP+ cells and evaluated after 8 days. The difference between vehicle and DOX-treated Tet-on LIN28B AML is not significant by paired t-test. Each paired data point reflects an independent AML specimen.

## SUPPLEMENTARY METHODS

### Mouse strains and treatments

The TetO_MLL-ENL and TetO_LIN28B lines were described previously (1-3). The *Lin28b*$^{flox}$ line was obtained from Jackson Laboratory (#023914). All procedures were performed according to an IACUC approved protocol at Washington University School of Medicine. Male and female mice were used in similar numbers for all experiments, with sample sizes estimated based on prior experience with these techniques. Experiments were non-blinded without randomization. Mice were housed in a standard pathogen free barrier facility, and all procedures were performed according to an IACUC approved protocol at Washington University School of Medicine. For transplantation experiments, mice were gamma irradiated with two doses of 550 cGy spaced by 3 hours for primary transplants or one dose of 600 cGy for secondary AML transplants. Cells were then injected into the retroorbital sinus. Where indicated, mice were fed doxycycline containing chow (200 ppm). For survival studies, mice were euthanized when they became moribund, and a diagnosis of AML was confirmed by flow cytometry and cytospin.

### Flow cytometry and colony formation assays

Cells were isolated, stained, analyzed and transplanted as previously described (4-6), using P0 liver or adult bone marrow as source material. Antibodies and gating strategies were described previously (4-6). Non-viable cells were excluded from analyses by 4',6-diamidino-2-phenylindone (DAPI) staining (1 μg/ml except as indicated below). Flow cytometry was performed on a BD FACSAria Fusion flow cytometer (BD

Biosciences). Colony formation assays were performed by culturing the indicated cells in Methocult M3434 media (Stem Cell Technologies). Colony morphologies were scored 12-14 days later. Annexin V assays were performed as previously described (4, 7).

**Western blots**

Thirty thousand cells were directly sorted into 10% Trichloracetic acid. The precipitates were pelleted, washed with acetone and solubilized for Western blotting as described previously (6) using antibodies to human LIN28B (Cell Signaling, 4196) or alpha Tubulin (Cell Signaling, 2144).

**AML cultures**

GFP+ AML cells were isolated by flow cytometry and cultured in StemSpan SFEM II (Stem Cell Technologies) supplemented with SCF (100 ng/mL) for 8 days with or without doxycycline (1 μg/mL). Cell counts were obtained using a hemacytometer. For morphology assays, GFP+ cells were cytospun and Wright-Giemsa stained.

**RNA-seq**

Total RNA was isolated from AML cells from the indicated groups using RNA-easy micro plus columns. Libraries were generated with Clontech SMART-seq kits and sequenced on a Novaseq S4. *Lin28b* levels were compared based on counts per million. RNA-seq data are available at Gene Expression Omnibus (Accession GSE253725).

## SUPPLEMENTARY REFERENCES


1. Ugale A, Norddahl GL, Wahlestedt M, Sawen P, Jaako P, Pronk CJ, et al. Hematopoietic stem cells are intrinsically protected against MLL-ENL-mediated transformation. Cell Rep. 2014;9(4):1246-55.

2. Okeyo-Owuor T, Li Y, Patel RM, Yang W, Casey EB, Cluster AS, et al. The efficiency of murine MLL-ENL-driven leukemia initiation changes with age and peaks during neonatal development. Blood Adv. 2019;3(15):2388-99.

3. Zhu H, Shyh-Chang N, Segre AV, Shinoda G, Shah SP, Einhorn WS, et al. The Lin28/let-7 axis regulates glucose metabolism. Cell. 2011;147(1):81-94.

4. Li Y, Kong W, Yang W, Patel RM, Casey EB, Okeyo-Owuor T, et al. Single-Cell Analysis of Neonatal HSC Ontogeny Reveals Gradual and Uncoordinated Transcriptional Reprogramming that Begins before Birth. Cell Stem Cell. 2020;27(5):732-47.

5. Li Y, Yang W, Wang HC, Patel RM, Casey EB, Denby E, et al. Basal type I interferon signaling has only modest effects on neonatal and juvenile hematopoiesis. Blood Adv. 2023;7(11):2609-21.

6. Porter SN, Cluster AS, Yang W, Busken KA, Patel RM, Ryoo J, et al. Fetal and neonatal hematopoietic progenitors are functionally and transcriptionally resistant to Flt3-ITD mutations. Elife. 2016;5:e18882.

7. Chen R, Okeyo-Owuor T, Patel RM, Casey EB, Cluster AS, Yang W, et al. Kmt2c mutations enhance HSC self-renewal capacity and convey a selective advantage after chemotherapy. Cell Rep. 2021;34(7):108751.
